# Chronic neuropeptide S treatment improves sleep architecture and reduces Aβ-related pathology in APP/PS1 mice

**DOI:** 10.3389/fneur.2026.1825666

**Published:** 2026-10-26

**Authors:** Yiru Zhao, Miaomiao Liu, Yanjun Zhou, Meng Cui, Nianshan Cai, Haiyi Wang, Guangming Liu, Hongxu Sun, Xiangpan Kong, Peng Zhao

**Affiliations:** 1 Department of Basic Medicine, Wuxi School of Medicine, Jiangnan University, Wuxi, China; 2 Jiangnan University Hospital, Jiangnan University, Wuxi, China

**Keywords:** Alzheimer’s disease, amyloid-β, microglia, neuropeptide S, NPSR, sleep

## Abstract

**Background:**

Sleep disturbances frequently occur in Alzheimer’s disease (AD). Although neuropeptide S (NPS) promotes wakefulness through the widely expressed NPS receptor (NPSR), its role in AD-related sleep dysregulation is unclear.

**Aims:**

To investigate the effects of chronic NPS administration on AD-related sleep disturbances beyond its acute wake-promoting effects.

**Methods:**

NPSR expression was examined in the hypothalamus and hippocampus of APPswe/PS1dE9 (APP/PS1) mice, a double-transgenic mouse model of AD. Mice received intracerebroventricular NPS (1 nmol/day) for 14 days. Sleep–wake states were classified as wakefulness, non-rapid eye movement (NREM) sleep, and rapid eye movement (REM) sleep. Sleep architecture and amyloid-β (Aβ)-related pathology were assessed in mice. Cell-associated Aβ levels, extracellular signal-regulated kinase (ERK) phosphorylation, and autophagy-related proteins were examined in BV2 cells. Sleep responses to subsequent intracerebroventricular Aβ₁–₄₂ administration were examined in NPS-treated APP/PS1 mice.

**Results:**

APP/PS1 mice had lower NPSR protein levels in the hypothalamus and hippocampus than wild-type (WT) controls. Compared with saline-treated APP/PS1 mice, NPS-treated mice showed increased NREM sleep, reduced wakefulness, and lower Aβ-related pathology. Changes in sleep amounts were most evident during the light phase. Subsequent Aβ₁–₄₂ administration increased wakefulness and reduced NREM sleep mainly during the dark phase. It also shortened NREM episodes during the light phase. In BV2 cells, NPS altered cell-associated Aβ levels and increased ERK phosphorylation. NPS treatment was also associated with higher LC3B-II and lower p62 levels. SCH772984 attenuated the NPS-associated changes in autophagy-related proteins and cell-associated Aβ.

**Conclusion:**

Chronic NPS administration improved sleep architecture and reduced Aβ-related pathology in APP/PS1 mice. The BV2 findings suggest possible involvement of ERK signaling and autophagy-related processes in cellular Aβ handling.

## Graphical abstract

**Figure:**
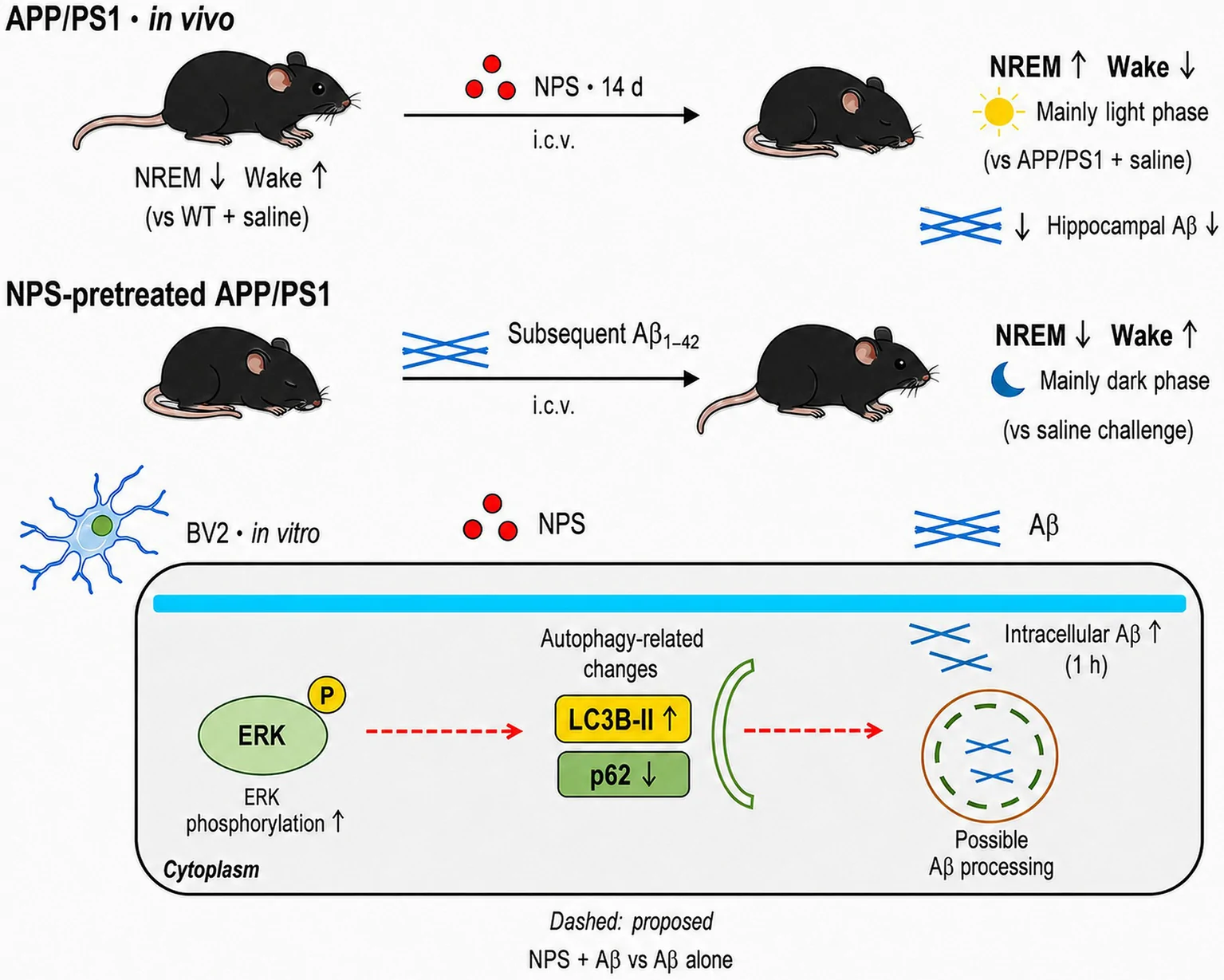
Chronic intracerebroventricular NPS administration for 14 days increased NREM sleep and reduced wakefulness, mainly during the light phase, and decreased hippocampal Aβ burden in APP/PS1 transgenic mice compared with saline treatment. Subsequent intracerebroventricular Aβ₁₋₄₂ administration in NPS-pretreated mice decreased NREM sleep and increased wakefulness, predominantly during the dark phase, compared with the additional saline injection. In Aβ₁₋₄₂-exposed BV2 microglial cells, NPS treatment was associated with increased cell-associated Aβ at 1 h, enhanced ERK phosphorylation, increased LC3B-II levels, and decreased p62 levels compared with Aβ₁₋₄₂ alone. Pharmacological ERK inhibition attenuated the NPS-associated changes in autophagy-related proteins and cell-associated Aβ. These in vitro findings suggest that ERK signaling and autophagy-related processes may be involved in cellular Aβ handling. Dashed arrows indicate proposed relationships rather than established causal mechanisms.

## 1 Introduction

Alzheimer’s disease (AD) is a progressive neurodegenerative disorder characterized by extracellular amyloid-β (Aβ) deposition and intracellular neurofibrillary tangles composed of hyperphosphorylated tau protein. Progressive impairment of memory and other cognitive functions affects patients’ independence and quality of life (1).

Sleep–wake states comprise wakefulness (W), non-rapid eye movement (NREM) sleep, and rapid eye movement (REM) sleep (2). Sleep–wake disturbances, including abnormalities in wakefulness, NREM sleep, and REM sleep, are common in patients with AD (3, 4). Related sleep–wake abnormalities have also been reported in transgenic mouse models (5–7). Sleep problems occur early in the course of AD. Individuals with amnestic mild cognitive impairment, many of whom may have early AD, show abnormalities in the electroencephalogram (EEG) during sleep, including fewer stage-2 sleep spindles and less time spent in deep NREM sleep (8). Clinically, sleep disturbances in AD include fragmented nighttime sleep, daytime sleepiness, and altered sleep–wake rhythms (3, 4). Poorer sleep quality has also been associated with amyloid deposition in cognitively unimpaired adults (9). These clinical observations, together with experimental findings, support a proposed bidirectional relationship between sleep disruption and AD-related pathology (10). Longitudinal evidence indicates that sleep disturbances, including insomnia symptoms and sleep-disordered breathing, are associated with an increased risk of subsequent cognitive impairment, mild cognitive impairment (MCI), and dementia, including AD (11–13).

Neuropeptide S (NPS) is a conserved 20-amino-acid neuropeptide primarily synthesized in the peri-locus coeruleus (peri-LC) and parabrachial nucleus of the brainstem (14, 15). It activates the Gαq-coupled NPS receptor (NPSR), which exhibits broad brain distribution with high density in the amygdala, thalamus, and prefrontal cortex (14, 16). The NPS–NPSR signaling axis regulates several neurobehavioral processes. It enhances cognition by facilitating hippocampal long-term potentiation (LTP) (17) and exerts anxiolytic effects (18). Moreover, the NPS–NPSR system plays an important role in regulating the sleep–wake cycle (14, 19, 20). Our previous study showed that 14-day intracerebroventricular (i.c.v.) administration of NPS reduced Aβ deposition and improved cognitive function in APPswe/PS1dE9 (APP/PS1) mice, a double-transgenic mouse model of AD (17). Acute i.c.v. administration of NPS (1 nmol) increases wakefulness and reduces NREM and REM sleep in rodents (19). This response is accompanied by activation of histaminergic and orexinergic neurons in the posterior hypothalamus (19). Experimental studies support reciprocal interactions between sleep–wake regulation and Aβ pathology (6, 21). Clinical studies have linked fragmented 24-h rest–activity rhythms to greater subsequent Aβ burden and cognitive decline (22, 23). Experimental studies in mice indicate that soluble Aβ oligomers can disrupt sleep. Acute intracerebroventricular administration of synthetic Aβ oligomers reduces NREM sleep and increases transitions between vigilance states, consistent with sleep fragmentation (24). These observations raise the question of how chronic NPS administration affects sleep in APP/PS1 mice and whether sleep changes are associated with changes in Aβ pathology.

In this study, we compared sleep–wake measures between saline-treated wild-type (WT) and APP/PS1 mice and evaluated the effects of chronic NPS treatment in APP/PS1 mice. We also examined sleep responses to subsequent Aβ₁–₄₂ administration in NPS-pretreated mice. Separate BV2 experiments examined cell-associated Aβ levels, ERK signaling, and autophagy-related proteins to explore a candidate cellular mechanism.

## 2 Results

### 2.1 Reduced NPSR expression in the hypothalamus and hippocampus of APP/PS1 mice

To examine the involvement of the NPS–NPSR system in AD-associated sleep disturbances, we first assessed NPSR expression in the hypothalamus and hippocampus. Western blotting showed lower NPSR protein levels in the hypothalamus (Figures 1A,B) and hippocampus (Figures 1C,D) of APP/PS1 mice than in WT controls. Consistent with the Western blot findings, immunohistochemical analysis showed fewer NPSR-positive cells per field in the hypothalamus (Figures 1E,F) and hippocampus (Figures 1G,H) of APP/PS1 mice. Together, the biochemical and histological measurements indicate reduced NPSR expression in both brain regions examined.

**Figure 1:**
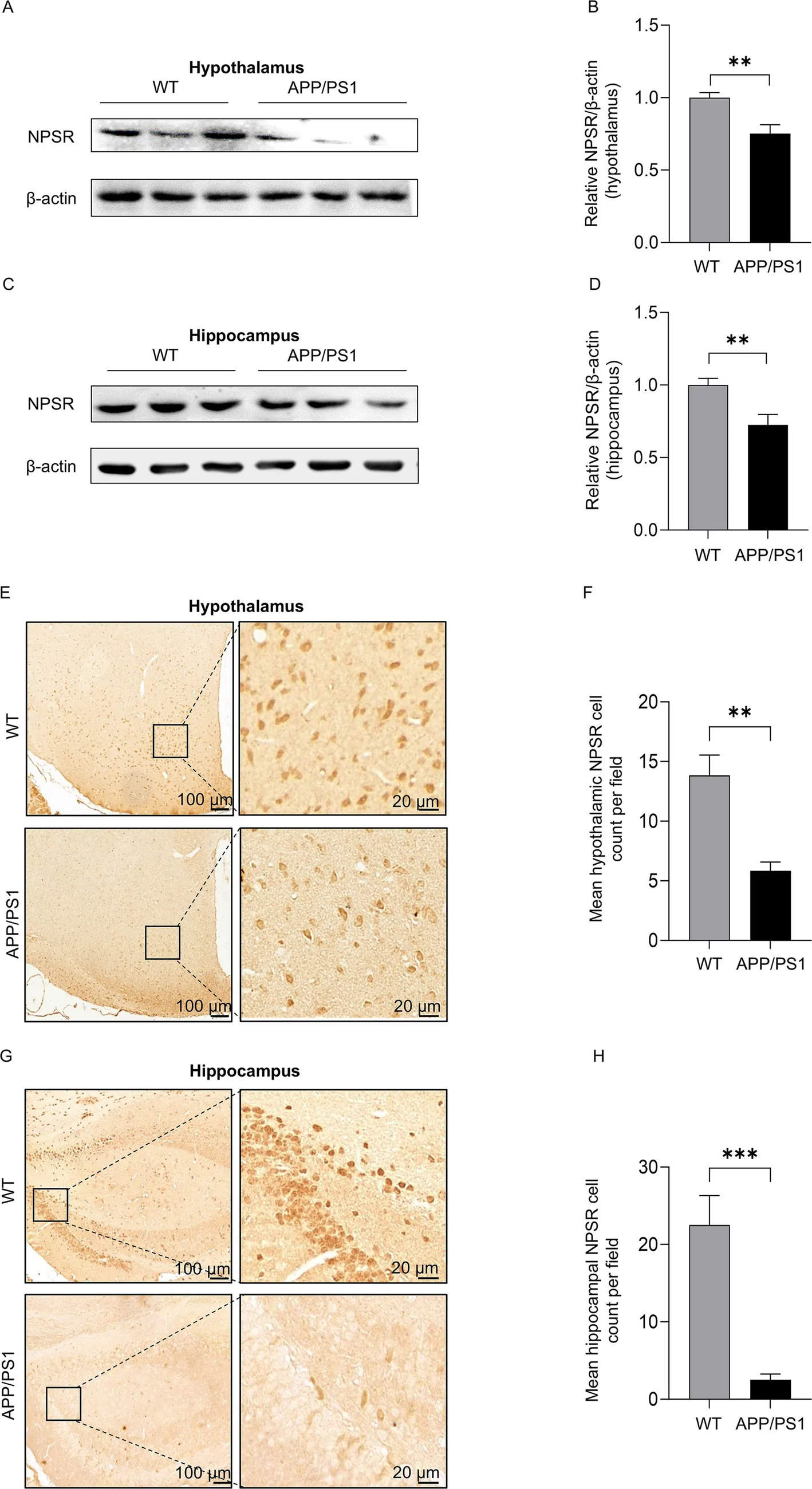
Reduced NPSR protein levels in the hypothalamus and hippocampus of APP/PS1 mice. **(A,B)** Representative immunoblots **(A)** and densitometric quantification **(B)** of hypothalamic NPSR protein levels in wild-type (WT) and APPswe/PS1dE9 (APP/PS1) mice. **(C,D)** Representative immunoblots **(C)** and densitometric quantification **(D)** of hippocampal NPSR protein levels. NPSR protein levels were normalized to β-actin. **(E)** Representative NPSR immunohistochemical images of the hypothalamus and corresponding enlarged fields. **(F)** Quantification of NPSR-positive cells per field in the hypothalamus. **(G)** Representative NPSR immunohistochemical images of the hippocampus and corresponding enlarged fields. **(H)** Quantification ofNPSR-positive cells per field in the hippocampus. Scale bars, 100 μm for low-magnification images and 20 μm for enlarged fields. Western blot data in panels **(B,D)** are presented as the mean ± SEM (*n* = 5 mice per group), and histological data in panels **(F,H)** are presented as the mean ± SEM (*n* = 6 mice per group). Statistical significance was assessed using an unpaired two-tailed Student’s t-test. **p < 0.01 and ***p < 0.001 versus the WT group.

### 2.2 Effects of chronic NPS treatment on NPSR expression and sleep–wake architecture in APP/PS1 mice

Representative NPSR immunoblots from the hypothalamus and hippocampus are shown in Figure 2A, B, respectively. Compared with saline-treated APP/PS1 mice, NPS-treated mice showed higher NPSR levels in the hypothalamus (Figure 2C) and hippocampus (Figure 2D).

**Figure 2:**
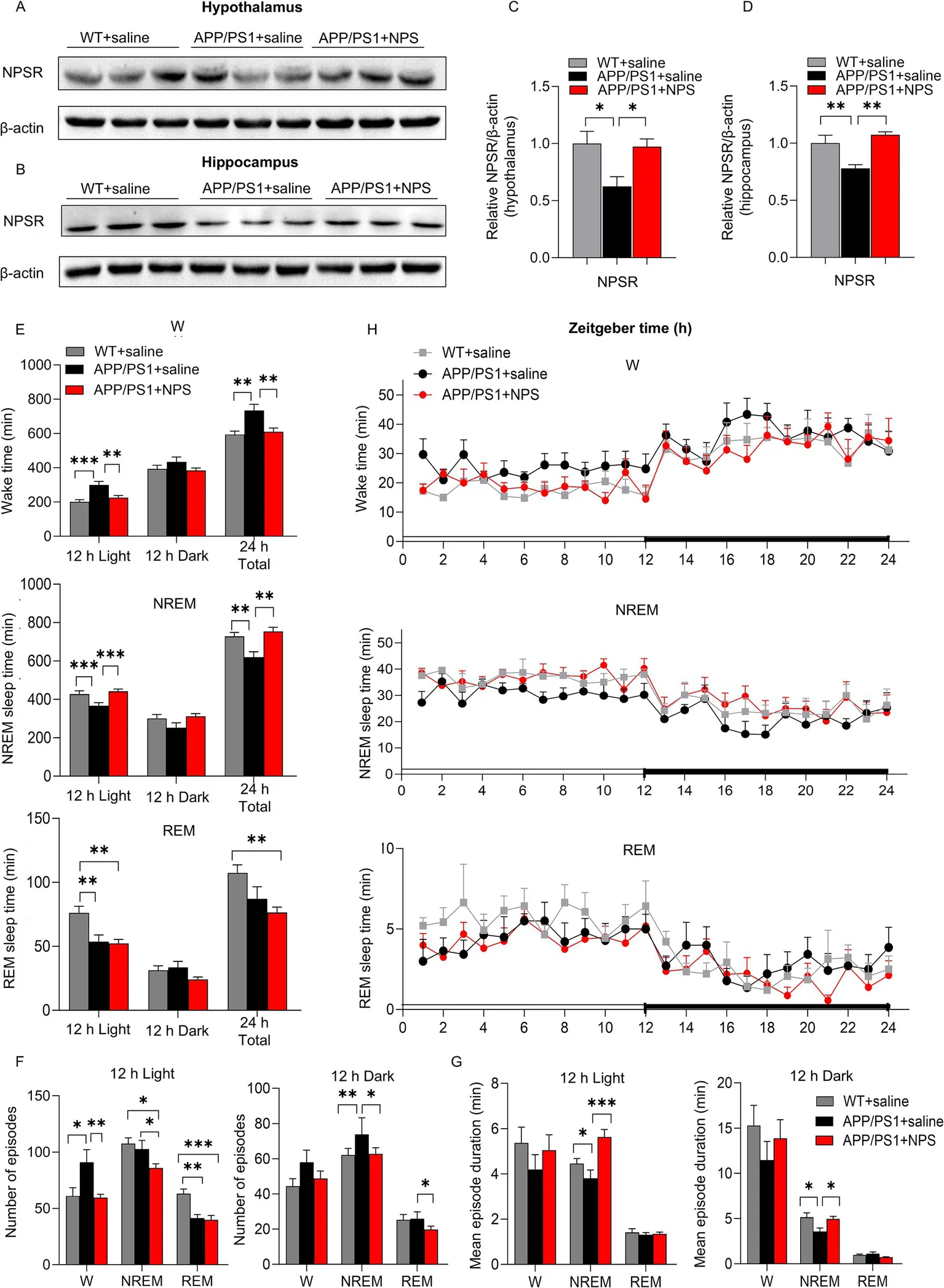
NPSR expression and sleep–wake architecture following chronic NPS treatment in APP/PS1 mice. The WT + saline, APP/PS1 + saline, and APP/PS1 + NPS groups are shown after 14 days of treatment. **(A,B)** Representative Western blots of NPSR in the hypothalamus **(A)** and hippocampus **(B)**. **(C,D)** Quantification of NPSR/β-actin in the hypothalamus **(C)** and hippocampus **(D)**. **(E)** Total time spent in wakefulness (W), non-rapid eye movement (NREM) sleep, and rapid eye movement (REM) sleep during the 12-h light phase, 12-h dark phase, and 24-h period. **(F,G)** Episode numbers **(F)** and mean episode durations **(G)** during the light and dark phases. **(H)** Hourly profiles of W, NREM sleep, and REM sleep over 24 h. Open and filled horizontal bars indicate the light and dark phases, respectively. Data are presented as the mean ± SEM (n = 6 mice per group). One-way ANOVA with Tukey’smultiple-comparisons test **(C–G)**; two-way repeated-measures ANOVA with Šídák’s multiple-comparisons test **(H)**. Brackets indicate the groups compared. *p < 0.05, **p < 0.01, and ***p < 0.001.

Sleep–wake measures were first compared between saline-treated WT and APP/PS1 mice. APP/PS1 mice spent more time awake and less time in NREM sleep during the light phase and over 24 h, and they spent less time in REM sleep during the light phase and over 24 h (Figure 2E). APP/PS1 mice also had more wake episodes and fewer REM episodes during the light phase, as well as more NREM episodes during the dark phase (Figure 2F). Mean NREM episode duration was shorter in APP/PS1 mice during both the light and dark phases (Figure 2G).

We next compared NPS-treated APP/PS1 mice with saline-treated APP/PS1 mice. After 14 days of NPS administration (1 nmol/day, i.c.v.), wakefulness was reduced during the light phase and over 24 h, whereas NREM sleep was increased during the light phase and over 24 h. REM sleep time did not differ significantly between the two APP/PS1 groups (Figure 2E). NPS-treated mice had fewer wake episodes during the light phase, fewer NREM episodes during both phases, and fewer REM episodes during the dark phase (Figure 2F). Mean NREM episode duration was longer during both the light and dark phases (Figure 2G). The corresponding hourly sleep–wake profiles are shown in Figure 2H.

### 2.3 Aβ-related pathology after NPS treatment and sleep changes following aβ₁–₄₂ administration

Representative Western blots of amyloid precursor protein (APP) and APP phosphorylated at threonine 668 (p-APP) in the hypothalamus and hippocampus are shown in Figures 3A,B, respectively. APP and p-APP levels were higher in saline-treated APP/PS1 mice than in saline-treated WT mice in both regions. Compared with saline-treated APP/PS1 mice, NPS-treated mice showed lower hypothalamic APP and p-APP levels (Figure 3C), as well as lower hippocampal APP and p-APP levels (Figure 3D). Representative 4G8 images from WT mice and the two APP/PS1 groups are shown in Figure 3E. Quantification in the two APP/PS1 groups showed a lower percentage of hippocampal 4G8-positive area after NPS treatment (Figure 3F). Thus, chronic NPS treatment was associated with lower APP and p-APP levels in the hypothalamus and hippocampus and with reduced 4G8-positive Aβ burden in the hippocampus.

**Figure 3:**
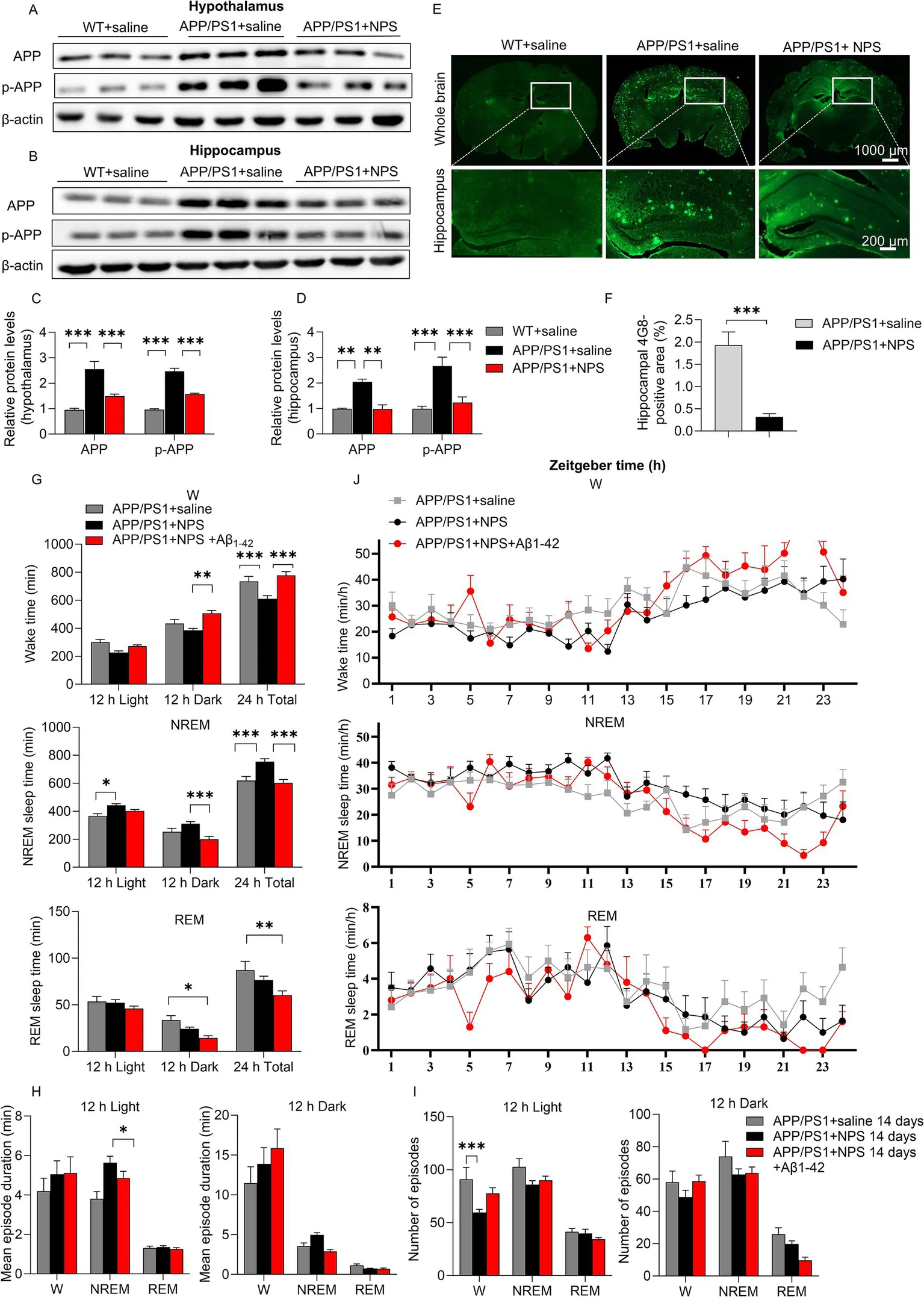
Aβ-related pathology after NPS treatment and sleep changes following Aβ₁–₄₂ administration. **(A,B)** Representative Western blots of APP and p-APP (Thr668) in the hypothalamus **(A)** and hippocampus **(B)**. **(C,D)** Quantification of APP and p-APP levels in the hypothalamus **(C)** and hippocampus **(D)**. β-actin served as the loading control. **(E)** Representative 4G8 immunofluorescence images of coronal brain sections and enlarged hippocampal regions from saline-treated WT mice, saline-treated APP/PS1 mice, and NPS-treated APP/PS1 mice. Green indicates 4G8-positive staining. Scale bars: 1000 μmfor overview images and 200 μm for enlarged views. **(F)** Hippocampal 4G8-positive area, expressed as a percentage of the field area, in saline- and NPS-treated APP/PS1 mice. For panels **(G–J)**, gray denotes saline-treated APP/PS1 mice, black denotes NPS-pretreated APP/PS1 mice given a subsequent saline injection, and red denotes NPS-pretreated APP/PS1 mice given a subsequent Aβ₁–₄₂ injection. **(G)** Total time spent in wakefulness (W), non-rapid eye movement (NREM) sleep, and rapid eye movement (REM) sleep during the 12-h light phase, 12-h dark phase, and 24-h period. **(H,I)** Mean episode durations **(H)** and episode numbers **(I)** during the light and dark phases. **(J)** Hourly profiles of W, NREM sleep, and REM sleep over 24 h. ZT0–12 and ZT12–24 correspond to the light and dark phases, respectively. Data are presented as the mean ± SEM (*n* = 6 mice per group). Unpaired two-tailed Student’s t-test **(F)**; one-way ANOVA with Tukey’s multiple-comparisons test **(C,D,G–I)**; two-way repeated-measures ANOVA with Šídák’s multiple-comparisons test **(J)**. Brackets indicate the groups compared. *p < 0.05, **p < 0.01, and ***p < 0.001.

To examine sleep responses to Aβ₁–₄₂ after chronic NPS treatment, we compared NPS-treated APP/PS1 mice receiving Aβ₁–₄₂ with those receiving a matched saline injection. APP/PS1 mice treated with saline for 14 days served as a reference group. During the dark phase, mice receiving Aβ₁–₄₂ spent more time awake and less time in NREM sleep than NPS-treated mice receiving the saline injection. The same differences were observed over 24 h (Figure 3G). REM sleep time did not differ significantly between these two groups.

During the light phase, mean NREM episode duration was shorter in mice receiving Aβ₁–₄₂ (Figure 3H). Episode counts and hourly sleep–wake profiles are shown in Figures 3I,J, respectively. Thus, Aβ₁–₄₂ administration after NPS treatment was associated with changes in wakefulness and NREM sleep amounts mainly during the dark phase, together with shorter NREM episodes during the light phase.

### 2.4 NPS reduces aβ plaque burden in APP/PS1 mice and alters cellular aβ handling and autophagy-related proteins in BV2 cells

To examine the spatial relationship between microglia and Aβ plaques following chronic NPS treatment, brain sections from saline- and NPS-treated APP/PS1 mice were co-stained for ionized calcium-binding adaptor molecule 1 (Iba1) and 4G8. Representative images showed Iba1-positive microglia in close proximity to 4G8-positive Aβ plaques in both groups (Figure 4A). Quantitative analysis showed that the mean number of 4G8-positive Aβ plaques per field was significantly lower in the NPS-treated group than in the saline-treated group (Figure 4B).

**Figure 4:**
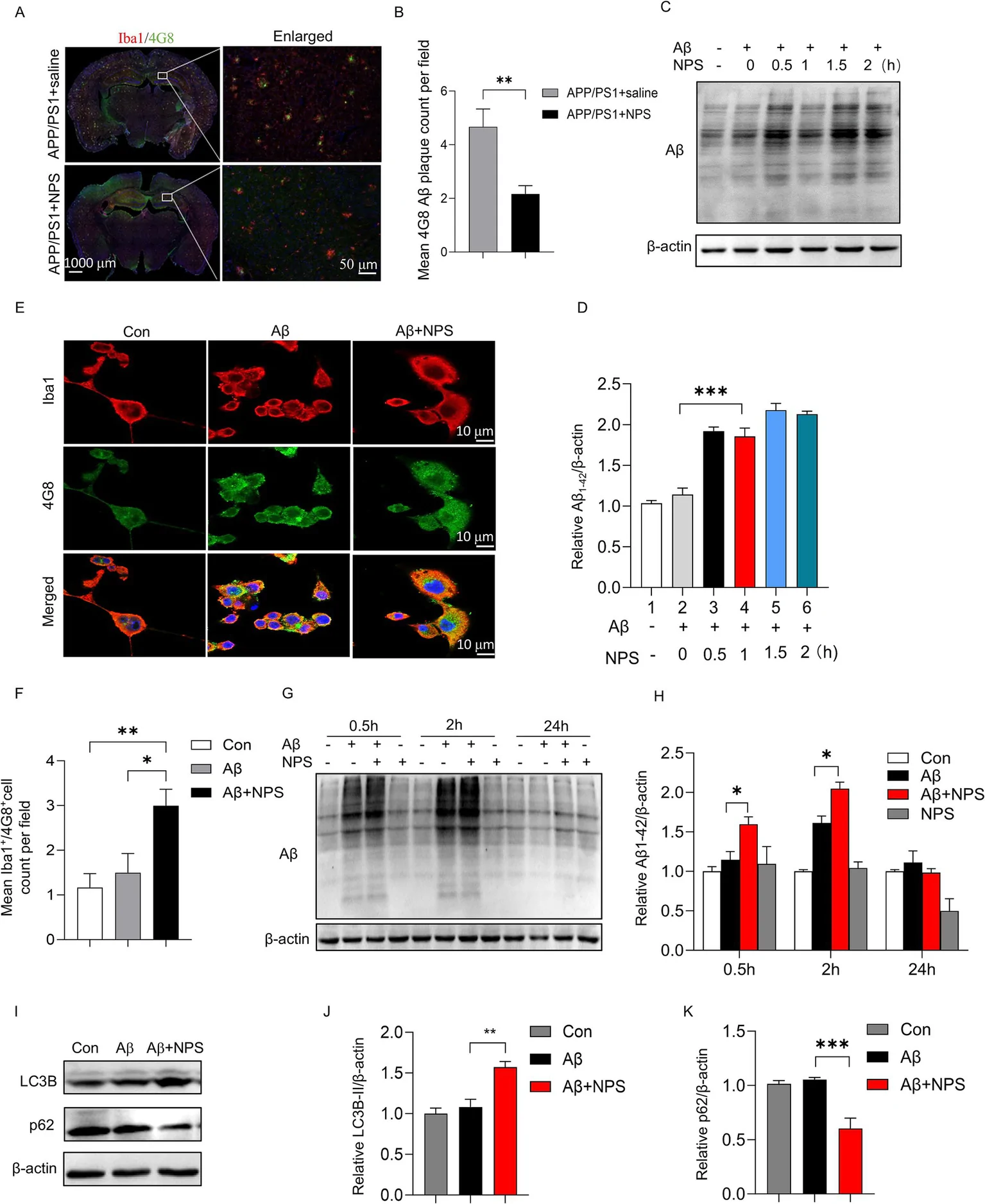
NPS reduces Aβ plaque burden and alters cellular Aβ handling and autophagy-related proteins. **(A)** Representative Iba1/4G8 immunofluorescence images of brain sections from saline- and NPS-treated APP/PS1 mice, with enlarged views of the boxed regions. **(B)** Mean numbers of 4G8-positive Aβ plaques per field. **(C,D)** Representative Western blots **(C)** and quantification **(D)** of cell-associated Aβ in BV2 cells under the indicated treatment conditions and NPS exposure times. **(E)** Representative Iba1/4G8 immunofluorescence images of untreated BV2 cells and cells exposed to Aβ₁–₄₂ alone or Aβ₁–₄₂ plus NPS for 2 h. **(F)** Mean numbers of Iba1/4G8 double-positive cells per field. **(G,H)** Representative Western blots **(G)** and quantification **(H)** of cell-associated Aβ in the washout experiment. The 24-h time point denotes 24 h after washout following a 2-h exposure. **(I)** Representative Western blots of LC3B and p62 in BV2 cells after 24 h under the indicated treatment conditions. **(J,K)** Quantification of LC3B-II **(J)** and p62 **(K)**. Aβ, LC3B-II, and p62 levels were normalized to β-actin. Data are presented as the mean ± SEM; n = 6 mice per group for B and *n* = 6 independent experiments for the cell assays. Unpaired two-tailed Student’s t-test (B); one-way ANOVA with Tukey’s multiple-comparisons test **(D,F,J,K)**; two-way ANOVA with Šídák’s multiple-comparisons test **(H)**. Brackets indicate the groups compared. *p < 0.05, **p < 0.01, and ***p < 0.001.

To examine early changes in cell-associated Aβ, BV2 cells were left untreated, exposed to Aβ₁–₄₂ alone, or exposed to Aβ₁–₄₂ together with NPS for the indicated periods. Compared with the Aβ₁–₄₂-only group, cell-associated Aβ levels were significantly higher after 1 h of NPS treatment, whereas no significant differences were detected at 0.5, 1.5, or 2 h (Figures 4C,D). The higher cell-associated Aβ level at 1 h indicates an early change in cellular Aβ handling but does not, by itself, distinguish increased uptake from reduced degradation.

BV2 cells were also co-stained for Iba1 and 4G8 after exposure to Aβ₁–₄₂ alone or Aβ₁–₄₂ plus NPS. The number of Iba1/4G8 double-positive cells per field was higher in the Aβ₁–₄₂ + NPS group than in the untreated and Aβ₁–₄₂-only groups (Figures 4E,F). These findings indicate increased cell-associated Aβ immunoreactivity but do not distinguish internalized Aβ from surface-associated Aβ or independently demonstrate increased phagocytosis.

A washout protocol was subsequently used to examine changes in cell-associated Aβ after removal of extracellular Aβ. Untreated cells and cells exposed to Aβ₁–₄₂ alone, Aβ₁–₄₂ plus NPS, or NPS alone were assessed after 0.5 and 2 h of exposure. After 2 h, extracellular Aβ was removed, and the cells were incubated in fresh medium for an additional 24 h before the final collection. Representative Western blots and the corresponding densitometric measurements are shown in Figures 4G,H. Before washout, cell-associated Aβ levels were higher in the Aβ₁–₄₂ + NPS group than in the Aβ₁–₄₂-only group after both 0.5 and 2 h of exposure. At the 24-h post-washout endpoint, cell-associated Aβ levels were similar between the two Aβ-exposed groups. These observations are consistent with cell-associated Aβ turnover after washout but do not establish that NPS accelerates Aβ degradation.

Autophagy is a lysosome-dependent pathway involved in the removal of long-lived proteins and protein aggregates (25, 26). LC3B-II associates with autophagosomal membranes, whereas SQSTM1/p62 acts as a selective autophagy cargo receptor and is itself degraded during autophagic processing; therefore, LC3B-II and p62 were assessed together as markers of autophagy-related changes (26). After 24 h of treatment, the Aβ₁–₄₂ + NPS group showed higher LC3B-II/β-actin and lower p62/β-actin levels than the Aβ₁–₄₂-only group (Figures 4I–K). These findings indicate changes in autophagy-related proteins. Because the measurements were obtained at a single endpoint without lysosomal inhibition, they do not establish increased autophagic flux (26).

### 2.5 ERK inhibition attenuates NPS-associated changes in autophagy-related proteins and cell-associated aβ in BV2 cells

Western blot analysis showed that the p-ERK/t-ERK ratio was lower in Aβ₁–₄₂-exposed BV2 cells than in untreated controls, whereas NPS co-treatment increased the p-ERK/t-ERK ratio relative to Aβ₁–₄₂ alone. In contrast, the p-p38/t-p38, p-JNK/t-JNK, and p-AKT/t-AKT ratios did not differ significantly among the groups (Figures 5A,B).

**Figure 5:**
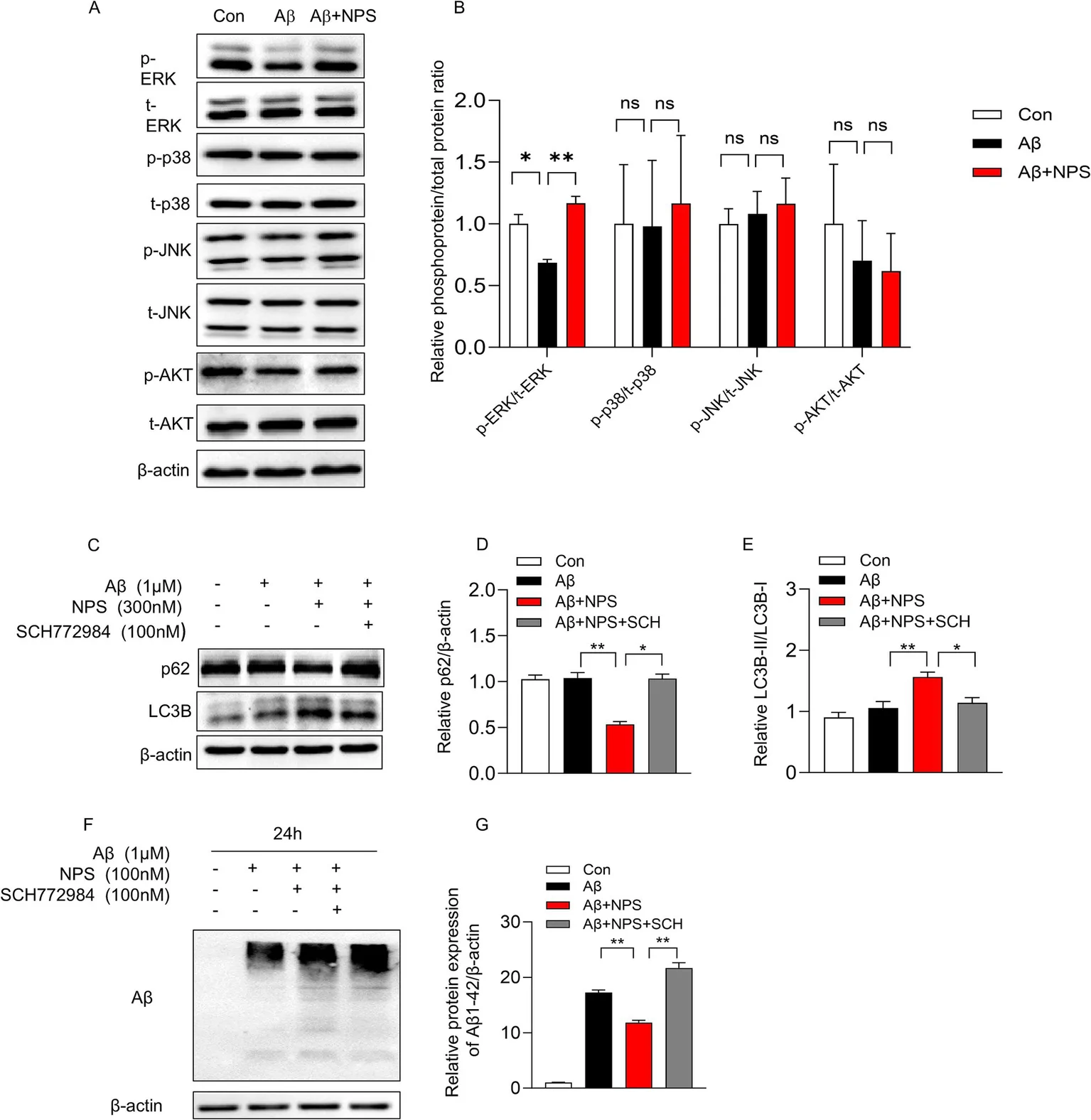
ERK inhibition attenuates NPS-associated changes in autophagy-related proteins and cell-associated Aβ in BV2 cells. **(A)** Representative Western blots of phosphorylated and total ERK, p38 MAPK, JNK, and AKT in untreated, Aβ₁–₄₂-treated, and Aβ₁–₄₂ + NPS-treated BV2 cells after 6 h. **(B)** Quantification of the p-ERK/t-ERK, p-p38/t-p38, p-JNK/t-JNK, and p-AKT/t-AKT ratios. **(C)** Representative Western blots of p62 and LC3B after 24 h under the indicated treatment conditions, with or without SCH772984. **(D)** Quantification of p62/β-actin. **(E)** Quantification of the LC3B-II/LC3B-I ratio. **(F,G)** Representative Western blots **(F)** and quantification of Aβ/β-actin **(G)** showing cell-associated Aβ after 24 h under the indicated treatment conditions. β-actin served as the loading control. Treatment groups and concentrations are indicated in the panels. Con denotes untreated cells; SCH denotes the ERK inhibitor SCH772984. Data are presented as the mean ± SEM (*n* = 6 independent experiments). One-way ANOVA with Tukey’s multiple-comparisons test was used for each protein ratio in panels **(B,D,E,G)**. Brackets indicate the groups compared. *p < 0.05 and **p < 0.01; ns, not significant.

In experiments using NPS at 300 nM, addition of SCH772984 (100 nM) increased p62 levels and reduced the LC3B-II/LC3B-I ratio relative to the Aβ₁–₄₂ + NPS group (Figures 5C–E). In separate experiments using NPS at 100 nM, cell-associated Aβ levels were lower with NPS co-treatment than with Aβ₁–₄₂ alone and were increased by SCH772984 relative to the Aβ₁–₄₂ + NPS group (Figures 5F,G). These pharmacological findings support possible ERK involvement in the cellular response to NPS.

## 3 Discussion

Chronic NPS treatment improved selected sleep measures and reduced amyloid-related pathology in APP/PS1 mice. Compared with saline-treated WT mice, saline-treated APP/PS1 mice showed increased wakefulness, reduced NREM sleep, and shorter NREM episodes. NPS treatment modified several of these abnormalities: wakefulness decreased and NREM sleep increased mainly during the light phase, while NREM episodes became longer during both phases. These changes were accompanied by lower hippocampal 4G8-positive area and lower APP and p-APP levels in the hypothalamus and hippocampus. Separate BV2 experiments identified changes in cellular Aβ levels, ERK phosphorylation, and autophagy-related proteins.

Sleep abnormalities have been reported in AD and amnestic MCI (3, 4, 8). Observational studies have linked sleep quality and rest–activity patterns to amyloid pathology and subsequent cognitive decline (9, 22, 27). These findings provide a clinical context for examining sleep and amyloid-related outcomes together. Acute central administration of NPS promotes wakefulness (14, 19), while genetic and circuit studies also implicate NPS–NPSR signaling in sleep regulation (20, 28). The increase in NREM sleep after chronic treatment in the present study differs from the acute wake-promoting response. However, treatment duration and disease background were not varied independently, so the reason for this difference remains uncertain. NPSR protein levels were lower in both brain regions examined, but measurements at a single age do not establish whether reduced receptor expression preceded or contributed to the sleep abnormalities.

The Aβ₁–₄₂ challenge experiment examined sleep responses to an additional intervention after chronic NPS treatment. Compared with NPS-pretreated mice receiving saline, those receiving Aβ₁–₄₂ showed increased wakefulness and reduced NREM sleep mainly during the dark phase, together with shorter NREM episodes during the light phase. This pattern only partly overlapped with the effects of chronic NPS, which were most evident during the light phase for sleep amounts. The experiment therefore shows that exogenous Aβ₁–₄₂ can alter sleep after NPS treatment, but does not establish a specific reversal of the NPS response. Without a corresponding Aβ₁–₄₂ challenge group lacking prior NPS treatment, it is also unclear whether the response depended on NPS pretreatment. The Aβ preparation contained DMSO, whereas the comparison group received saline alone; therefore, vehicle-related effects cannot be excluded.

The reduction in amyloid-related pathology cannot be attributed to microglial clearance alone. Although Iba1/4G8 co-staining showed fewer 4G8-positive plaques per field after NPS treatment and illustrated the spatial proximity between Iba1-positive microglia and plaques, it did not directly quantify Aβ internalization or establish that microglia were required for the treatment effect. Lower APP and p-APP levels also leave open the possibility of altered Aβ production. Changes in sleep architecture and other clearance processes may also have contributed, because sleep has been shown to facilitate brain interstitial solute and Aβ clearance (29); however, their respective roles were not tested in the present study.

The BV2 experiments provide evidence for a cellular response to NPS. Immunofluorescence analysis showed more Iba1/4G8 double-positive cells per field after NPS co-treatment, and Western blotting showed higher cell-associated Aβ after 1 h of NPS exposure. However, these measurements do not distinguish intracellular uptake from surface-associated Aβ, altered degradation, release, or differences in cell density. After washout, Aβ levels declined and were similar between the two Aβ-exposed groups at the final endpoint. Higher LC3B-II and lower p62 levels are consistent with changes in autophagy-related processes, but endpoint measurements without lysosomal inhibition cannot establish increased autophagic flux (26).

The increased p-ERK/t-ERK ratio and the effects of SCH772984 support possible ERK involvement in the BV2 response. However, ERK inhibition does not selectively test autophagy. The autophagy-marker and Aβ experiments also used different NPS concentrations, 300 nM and 100 nM, respectively. They therefore do not directly establish an ERK–autophagy–Aβ degradation sequence under the same treatment conditions. Whether these cellular responses contribute to the changes observed in mice remains unresolved.

Several additional limitations affect interpretation. The absence of a WT + NPS group prevents assessment of whether the chronic sleep response is specific to the APP/PS1 model. The animal experiments were restricted to 8-month-old males, and the cell experiments used an immortalized BV2 cell line rather than primary microglia. The histological measurements were based on selected coronal sections and fields and did not directly assess microglial functional phenotype or the intracellular localization of Aβ. In addition, Iba1 is a microglial marker (30), but Iba1 staining alone does not establish an activation phenotype. The Iba1/4G8 double-positive-cell count in BV2 cultures may also be influenced by cell density and cannot separately resolve Aβ uptake, degradation, and release. These limitations should be considered when extending the findings to other ages, females, or microglia in the adult brain.

In conclusion, chronic i.c.v. NPS treatment improved selected aspects of sleep architecture and reduced amyloid-related pathology in APP/PS1 mice. The BV2 experiments suggest possible involvement of ERK signaling and autophagy-related processes in cellular Aβ handling. Their contribution to the sleep and pathological effects of NPS *in vivo* remains to be established.

## 4 Materials and methods

### 4.1 BV2 cell line

The BV2 microglial cell line was purchased from Procell Life Science & Technology Co., Ltd. (Wuhan, China; Cat. No. CL-0493). Cells were cultured in high-glucose Dulbecco’s modified Eagle’s medium (DMEM; HyClone) supplemented with 10% fetal bovine serum (FBS; HyClone) and 1% penicillin–streptomycin (Sigma). Cells were maintained at 37 °C in a humidified atmosphere containing 5% CO₂, and only cells at passage numbers not exceeding 5 were used for experiments. For the indicated experiments, cells were seeded in 6-well plates.

### 4.2 BV2-cell exposure and washout experiments

For the early-exposure experiments shown in Figures 4C, D, BV2 cells were exposed to Aβ₁–₄₂ (1 μM) alone or together with NPS for 0.5, 1, 1.5, or 2 h before collection for Western blot analysis.

For the washout experiments shown in Figures 4G,H, BV2 cells were exposed to Aβ₁–₄₂ (1 μM) alone or together with NPS. Samples were collected after 0.5 and 2 h of exposure to assess cell-associated Aβ before washout. After the 2-h exposure, extracellular Aβ was removed and the medium was replaced with fresh medium. Cells were then incubated for an additional 24 h before the final collection. Thus, the final endpoint represented 24 h after washout, rather than 24 h after the initial addition of Aβ.

### 4.3 Animals

Male APPswe/PS1dE9 double-transgenic mice and their wild-type littermates, all aged 8 months, were obtained from the Model Animal Center of Nanjing University [license No. SCXK (Su) 2015–001]. Mice were group-housed in standard cages (41 × 16 × 18 cm) under controlled conditions: 23 ± 2 °C and 55 ± 10% relative humidity under a 12-h light/dark cycle, with lights on at 07:00 (Zeitgeber time [ZT] 0) and off at 19:00. Food and water were available ad libitum. All procedures were approved by the Jiangnan University Animal Ethics Committee (Protocol No. JN. No20211215m0880530[586]).

### 4.4 Aβ₁–₄₂ and NPS preparation

Aβ₁–₄₂ was purchased from AnaSpec (Fremont, CA, USA). For intracerebroventricular administration, a stock solution was prepared at 5 μg/μL in sterile saline containing 0.1% dimethyl sulfoxide (DMSO). The stock solution was diluted to a working concentration of 3 μg/μL and incubated at 37 °C for 4 days before use. Each injection contained 6 μg of Aβ₁–₄₂ in a volume of 2 μL. The mouse NPS (SFRNGVGSGAKKTSFRRAKQ) was purchased from Cellmano Biotech Ltd. (Hefei, China). For intracerebroventricular administration, NPS was dissolved in sterile saline at a concentration of 0.5 mM, providing 1 nmol in an injection volume of 2 μL. For cell experiments, Aβ₁–₄₂ was dissolved in hexafluoroisopropanol (HFIP; 1 mg/mL) with sonication for 10 min. HFIP was evaporated to form a peptide film, which was subsequently dissolved in DMSO. Aβ₁–₄₂ was added to the culture medium at a final concentration of 1 μM.

### 4.5 Surgeries

All surgeries were performed under sodium pentobarbital anesthesia (50 mg/kg, i.p.) using a stereotaxic apparatus (Narishige SR-6R). Intracerebroventricular cannulation and drug administration: A guide cannula (24 G, 9 mm in length) was stereotaxically implanted with its tip positioned in the right lateral ventricle at 0.6 mm posterior to bregma, 1.0 mm lateral to the midline on the right, and 2.5 mm ventral to the horizontal plane through bregma, with reference to the mouse brain atlas of Paxinos and Franklin (2001). Following 7 days of postoperative recovery, mice received daily i.c.v. injections of NPS (1 nmol in 2 μL) or saline for 14 days. Injections were administered at 20:00 during the dark phase at a rate of 1 μL/min. Mice were acclimated to the recording chambers for 3 days during the treatment period. Following the 14-day treatment, sleep–wake activity was recorded continuously from 07:00 on day 15 to 07:00 on day 16, covering one complete light–dark cycle. The chronic-treatment experiment included saline-treated WT mice, saline-treated APP/PS1 mice, and NPS-treated APP/PS1 mice. Comparisons between the two saline-treated groups were used to describe the sleep phenotype associated with the APP/PS1 model. Comparisons between the two APP/PS1 groups were used to assess the effects of NPS.

In the subsequent Aβ₁–₄₂ challenge experiment, APP/PS1 mice that had received NPS for 14 days were given a single intracerebroventricular injection of Aβ₁–₄₂ (6 μg in 2 μL, prepared as described above) or 2 μL of sterile saline 24 h before polysomnographic recording. The saline comparison injection matched the Aβ₁–₄₂ injection in volume, procedure, and timing but did not contain the 0.1% DMSO present in the Aβ₁–₄₂ preparation. APP/PS1 mice treated with saline for 14 days served as a reference group.

Electrode implantation: Four screw electrodes were implanted through the skull to contact the dura for EEG recording. Two silver-plated electrodes were inserted into the nuchal muscles for electromyography (EMG) recording. Electrode leads were soldered to a 6-pin connector secured to the skull with dental cement.

### 4.6 Polygraphic recordings and sleep–wake state classification

Following surgical recovery, mice undergoing sleep–wake cycle recordings were individually housed in soundproof, ventilated chambers equipped with a low-torque commutator permitting unrestricted movement and ad libitum access to resources. EEG and EMG signals were amplified 2000-fold using a Model 3,500 system (A-M Systems) with bandpass filtering (0.5–60 Hz for EEG; 30–300 Hz for EMG), then digitized at 256 Hz and 128 Hz, respectively, via a CED 1401 MK II interface (Cambridge Electronic Design). Behavioral states were continuously monitored by infrared videography. Polygraphic recordings were visually scored in consecutive 30-s epochs using the Spike2 sleep-scoring script (CED), with fast Fourier transform (FFT)-assisted spectral analysis (19, 31). W was characterized by low-amplitude, high-frequency, desynchronized EEG activity, with predominant activity in the 14.5–60 Hz range and sustained or phasic EMG activity. NREM sleep was defined by high-amplitude, low-frequency EEG activity dominated by delta activity (0.5–4.0 Hz), together with reduced EMG tone. REM sleep was identified by low-amplitude EEG activity dominated by theta activity (4.5–8.5 Hz) and minimal or absent tonic EMG activity, with occasional muscle twitches. Each 30-s epoch was assigned to the state occupying the greatest proportion of that epoch. No fixed absolute EEG/EMG amplitude or spectral-power cutoff was applied; classification was based on the combined assessment of EEG waveform, frequency-band predominance, EMG tone, and video-recorded behavior.

### 4.7 Immunohistochemistry

Immunohistochemistry was performed as described previously (32). Briefly, paraffin-embedded brain sections were deparaffinized in xylene and rehydrated through graded ethanol series. Following antigen retrieval and endogenous peroxidase blocking, sections were blocked with 5% bovine serum albumin (BSA). Sections were then incubated overnight at 4 °C with rabbit anti-NPSR primary antibody (1:1000; Abcam ab92425). Detection was performed using a horseradish peroxidase (HRP)-conjugated secondary antibody kit (ASP Rabbit & Mouse HRP Kit, CW2069S; KangWei Biotech) with DAB-nickel ammonium sulfate substrate to generate insoluble chromogenic complexes. Immunohistochemical images were acquired using identical imaging settings for all groups. Six mice per group were included in the histological analysis. Three coronal sections were analyzed from each mouse, and three randomly selected non-overlapping fields were assessed in each section. NPSR-positive cells were counted in each field, and the mean of the nine fields was calculated to obtain one value for each mouse. Thus, the mouse, rather than an individual section or field, was treated as the independent experimental unit.

### 4.8 Immunofluorescence assay

For brain-tissue immunofluorescence, mice were perfused with saline followed by 4% paraformaldehyde. Brains were post-fixed overnight, cryoprotected sequentially in 10%, 20%, and 30% sucrose, and cut into 30-μm coronal sections. Sections were permeabilized with 0.2% Triton X-100 in PBS for 10 min and blocked with 5% BSA at 37 °C for 1 h. For the 4G8 staining shown in Figure 3E, sections were incubated with mouse monoclonal anti-β-amyloid antibody (clone 4G8; Merck Millipore, catalog no. MAB1561, 1:300) at 4 °C for 24 h, followed by Fluoro 488-conjugated AffiniPure donkey anti-mouse IgG (H + L) (Boster Biological Technology, Wuhan, China; catalog no. BA1145, 1:300) at 37 °C for 1 h. Sections were mounted using an antifade medium containing DAPI and imaged with a Zeiss LSM880 confocal microscope.

For the tissue co-staining shown in Figure 4A, sections were incubated with rabbit anti-Iba1 antibody (Wako, catalog no. 019–19,741, 1:300) and mouse monoclonal anti-β-amyloid antibody (clone 4G8; Merck Millipore, catalog no. MAB1561, 1:300). Fluoro 594-conjugated AffiniPure donkey anti-rabbit IgG (H + L) (Boster Biological Technology, Wuhan, China; catalog no. BA1147, 1:300) and Fluoro 488-conjugated AffiniPure donkey anti-mouse IgG (H + L) (Boster Biological Technology, Wuhan, China; catalog no. BA1145, 1:300) were used as the corresponding secondary antibodies.

The remaining staining procedures followed the tissue protocol described above.

For the BV2-cell co-staining shown in Figure 4E, untreated cells and cells exposed to Aβ₁–₄₂ (1 μM) alone or Aβ₁–₄₂ plus NPS for 2 h were incubated with rabbit anti-Iba1 antibody (Wako, catalog no. 019–19,741, 1:200) and mouse monoclonal anti-β-amyloid antibody (clone 4G8; Merck Millipore, catalog no. MAB1561, 1:200). Fluoro 594-conjugated AffiniPure donkey anti-rabbit IgG (H + L) (Boster Biological Technology, Wuhan, China; catalog no. BA1147, 1:200) and Fluoro 488-conjugated AffiniPure donkey anti-mouse IgG (H + L) (Boster Biological Technology, Wuhan, China; catalog no. BA1145, 1:200) were used as the corresponding secondary antibodies. Cells were incubated with the secondary antibodies at 37 °C for 1 h. DAPI-containing antifade mounting medium was applied before mounting.

Quantification of tissue immunofluorescence was performed using ImageJ. Six mice per group were analyzed. Three coronal brain sections containing the hippocampus were analyzed from each mouse, and three randomly selected non-overlapping fields were acquired from each section. For Figure 3F, the 4G8-positive area in each field was expressed as a percentage of the total field area. For Figure 4B, the number of 4G8-positive Aβ plaques was counted in each field. Values obtained from the nine fields were averaged to generate one value for each mouse, and the mouse was treated as the independent experimental unit (n = 6 mice per group). For the BV2-cell immunofluorescence analysis shown in Figures 4E,F, Iba1/4G8 double-positive cells were counted per field using ImageJ. The mean value from the analyzed fields in each independent experiment was used as one biological replicate (n = 6 independent experiments). Iba1 was used as a microglial marker; Iba1 staining alone was not interpreted as evidence of microglial activation or a specific functional phenotype.

### 4.9 Western blot

Following polysomnographic recordings, mice were euthanized by decapitation. Hippocampi and hypothalami were rapidly dissected, homogenized, and centrifuged to obtain protein lysates. Protein concentration was quantified using a BCA assay (Thermo Scientific 23,227). Equal amounts (20 μg) of protein were resolved on 6–12% gradient SDS-PAGE gels and transferred to nitrocellulose membranes. Membranes were blocked with 5% non-fat milk and incubated overnight at 4 °C with the indicated primary antibodies. Rabbit anti-NPSR (Abcam, ab92425, 1:1000) and rabbit anti-phospho-APP (Thr668; clone D90B8; Cell Signaling Technology, 6,986, 1:1000) antibodies were used. Rabbit polyclonal antibodies against APP (AF7687), Aβ₁–₄₂ (AF0019), SQSTM1/p62 (AF5384), LC3B (AF4650), ERK1/2 (AF6240), phospho-ERK1/2 (Thr202/Tyr204; AF1015), p38 MAPK (AF6456), phospho-p38 MAPK (Thr180/Tyr182; AF4001), JNK1/2/3 (AF6318), phospho-JNK1/2/3 (Thr183/Tyr185; AF3318), pan-AKT1/2/3 (AF6261), and phospho-AKT1/2/3 (Ser473; AF0016) were obtained from Affinity Biosciences (Changzhou, China) and used at a dilution of 1:1000.

A rabbit anti-β-actin antibody (Affinity Biosciences, AF7018, 1:5000) was used as the loading control. After washing with Tris-buffered saline containing Tween 20 (TBST), membranes were incubated with HRP-conjugated goat anti-rabbit IgG (H + L) secondary antibody (Thermo Fisher Scientific, 31,460, 1:10000) for 2 h at room temperature. Membranes were then washed with TBST. Immunoreactive bands were detected using enhanced chemiluminescence (Thermo Scientific, 34,580), and band intensities were quantified using ImageJ. Protein signals were normalized to β-actin where indicated. Phosphorylated proteins were normalized to their corresponding total-protein signals (p-ERK/t-ERK, p-p38/t-p38, p-JNK/t-JNK, and p-AKT/t-AKT), and the LC3B-II/LC3B-I ratio was calculated as specified in the corresponding figure legends.

### 4.10 Statistical analysis

All data are presented as the mean ± standard error of the mean (SEM) and were analyzed using SPSS version 12.0 (SPSS Inc., Chicago, IL, USA). Figures were generated using GraphPad Prism version 8.0.2 (GraphPad Software Inc., La Jolla, CA, USA). Comparisons between two independent groups were performed using an unpaired two-tailed Student’s t-test. Comparisons among three or more independent groups were performed using ordinary one-way analysis of variance (ANOVA), followed by Tukey’s multiple-comparisons test. The washout time-course experiment shown in Figures 4G,H was analyzed using ordinary two-way ANOVA, with treatment and time as factors, followed by Šídák’s multiple-comparisons test. Hourly sleep–wake data repeatedly recorded from the same animals were analyzed using two-way repeated-measures ANOVA, with group as the between-subject factor and time as the within-subject factor, followed by Šídák’s multiple-comparisons test. Differences were considered statistically significant at *p* < 0.05. Significance levels are indicated as *p < 0.05, **p < 0.01, and ***p < 0.001.

## Data Availability

The original contributions presented in the study are included in the article/supplementary material, further inquiries can be directed to the corresponding author.
